# Lack of association between NAT2 polymorphism and prostate cancer risk: a meta-analysis and trial sequential analysis

**DOI:** 10.18632/oncotarget.19023

**Published:** 2017-07-05

**Authors:** Feng Wang, Zhiqiang Qin, Shuhui Si, Jingyuan Tang, Lingyan Xu, Haoxiang Xu, Ran Li, Peng Han, Haiwei Yang

**Affiliations:** ^1^ Department of Radiation Oncology, The First Affiliated Hospital of Nanjing Medical University, Nanjing, 210009, China; ^2^ State Key Laboratory of Reproductive Medicine, Department of Urology, The First Affiliated Hospital of Nanjing Medical University, Nanjing, 210029, China; ^3^ Research Division of Clinical Pharmacology, The First Affiliated Hospital of Nanjing Medical University, Nanjing, 210009, China; ^4^ Department of Oncology, The First Affiliated Hospital of Nanjing Medical University, Nanjing, 210009, China

**Keywords:** NAT2*4, gene polymorphism, prostate cancer, meta-analysis

## Abstract

Previous studies have investigated the association between NAT2 polymorphism and the risk of prostate cancer (PCa). However, the findings from these studies remained inconsistent. Hence, we performed a meta-analysis to provide a more reliable conclusion about such associations. In the present meta-analysis, 13 independent case-control studies were included with a total of 14,469 PCa patients and 10,689 controls. All relevant studies published were searched in the databates PubMed, EMBASE, and Web of Science, till March 1st, 2017. We used the pooled odds ratios (ORs) with 95% confidence intervals (CIs) to evaluate the strength of the association between NAT2*4 allele and susceptibility to PCa. Subgroup analysis was carried out by ethnicity, source of controls and genotyping method. What's more, we also performed trial sequential analysis (TSA) to reduce the risk of type I error and evaluate whether the evidence of the results was firm. Firstly, our results indicated that NAT2*4 allele was not associated with PCa susceptibility (OR = 1.00, 95% CI= 0.95–1.05; *P* = 0.100). However, after excluding two studies for its heterogeneity and publication bias, no significant relationship was also detected between NAT2*4 allele and the increased risk of PCa, in fixed-effect model (OR = 0.99, 95% CI= 0.94–1.04; *P* = 0.451). Meanwhile, no significant increased risk of PCa was found in the subgroup analyses by ethnicity, source of controls and genotyping method. Moreover, TSA demonstrated that such association was confirmed in the present study. Therefore, this meta-analysis suggested that no significant association between NAT2 polymorphism and the risk of PCa was found.

## INTRODUCTION

Prostate cancer (PCa) is considered as the second most leading cause of cancer-related deaths among men worldwide [1, 2]. PCa is a multifactorial disease caused by complex interactions between a series of potential risk factors, such as environment, ethnicity, age, and genetic factors. However, the accurate etiology of PCa remained unclear. Nowadays, epidemiologic studies have pointed that several environmental factors such as smoking, ultra-violet radiation, and diet, including meat and fat intake, possibly had an increased risk of developing PCa [3, 4]. Nevertheless, a large number of candidate genes responsible for PCa have been identified. Genetic factors, particularly single-nucleotide genetic polymorphisms (SNPs), might prove to be reliable in predicting the genetic risk of PCa, thus possibly contributing to the primary prevention of this condition [5].

N-acetyltransferase 2 (NAT2) gene is clustered on the short arm of chromosome 8 (8q22) and encodes a phase II xenobioticmetabolizing enzyme [6, 7]. Besides, NAT2 is essentially involved in heterocyclic amines, hydrazines and aromatic metabolites [8, 9]. The alteration of NAT2 acetylator status caused by polymorphosms in NAT2 gene may induce the decrease of enzyme activity and the absence of efficiency in detoxification, thus contributing to increasing cancer susceptibility [10]. There are two major NAT2 phenotypes, including rapid acetylator and slow acetylator. Until now, over sixty NAT2 genetic variants have been discovered in humans, in which NAT2*4 is deemed to the most common allele related to rapid acetylation, and has been designated ‘‘wild-type’’ in history [11–13]. In addition, mutant homozygotes in NAT2 gene are classified as slow acetylator phenotype, while wild-type homozygotes and heterozygotes are categorized into rapid acetylator phenotype.

To date, a number of studies have been performed to elucidate the association between NAT2 genetic polymorphism and susceptibility to PCa. Meanwhile, some studies have demonstrated that people with low NAT2 activity have a higher risk of developing PCa, compared to those with high NAT2 activity [14]. However, the results remained inconsistent or even contrary. Therefore, further assessment is need to be proved to the real association and whether or not rapid acetylation is a risk factor for the development of PCa. Hence, we aimed to conduct a meta-analysis with all accessible case-control studies and trial sequential analysis (TSA) to gain the more precise evidence for the relationship between NAT2 genetic polymorphism and PCa risk.

## RESULTS

### Characteristics of the studies

**Figure 1:**
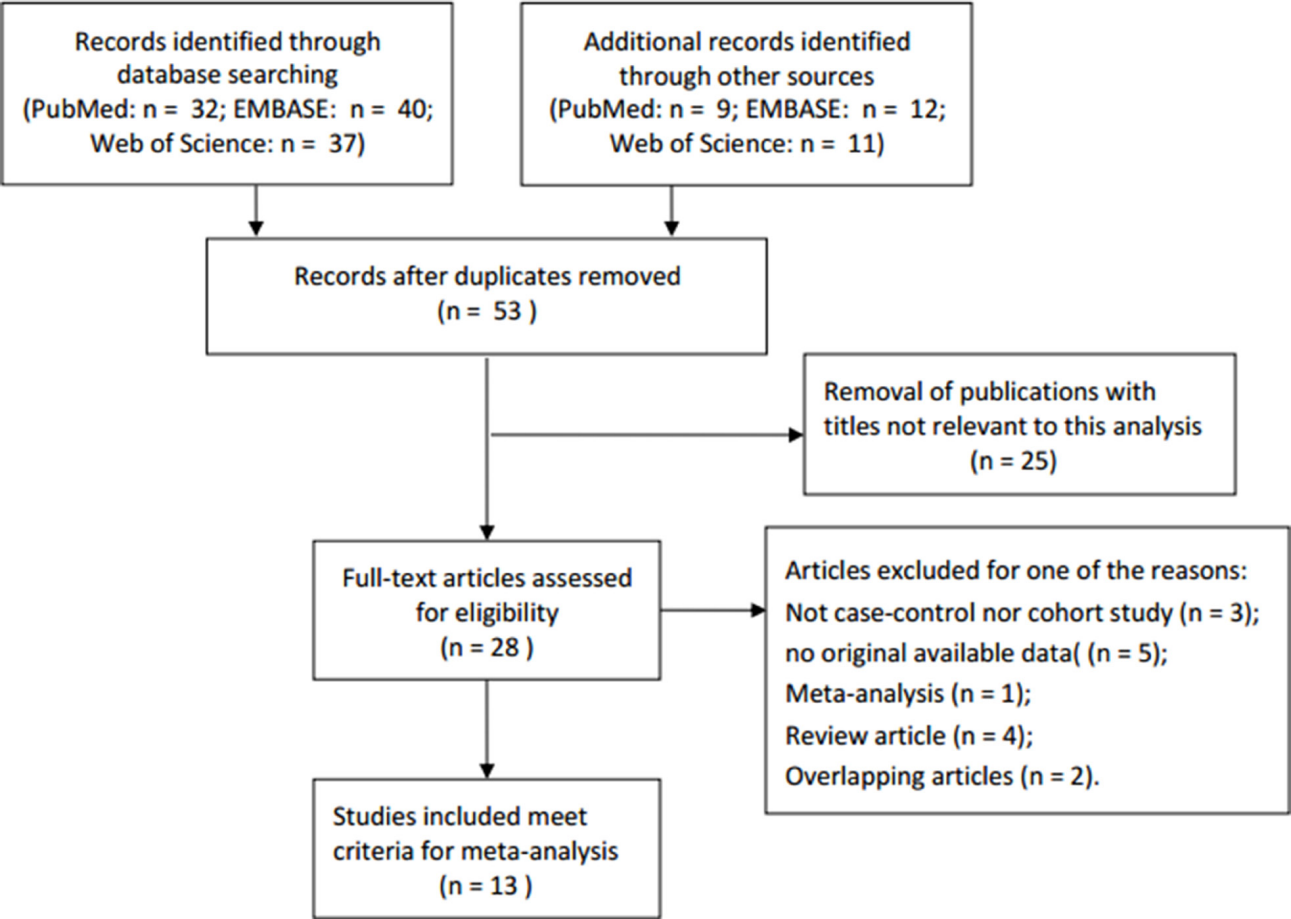
Flow diagram of literature search and selection process

Overall, thirteen independent case-control studies were included with a total of 14,469 PCa patients and 10,689 controls in the present meta-analysis [22–34]. Table 1 showed the detailed characteristics and genotype distribution of the selected studies reported the association between NAT2*4 allele and PCa risk. The process of literature search and selection with specification of reasons was listed in Figure 1. Among these previous studies, ten studies were conducted on Caucasian populations, one was Asians, one was mixed races, and the remaining study was conducted in African population. Furthermore, we consisted of 9 population-based studies and 4 hospital-based studies, in order to distinguish between different sources of control group. Diverse genotyping methods were included: TaqManSNP (TaqMan), polymerase chain reaction (PCR), PCR-restriction fragment length polymorphism (PCR-RFLP).

**Table 1:** Characteristics of studies that investigated the association between NAT2 polymorphism and prostate cancer risk

Author	Year	Country	Ethnicity	SOC	Genotyping methods	case	control	Non-NAT2*4 of case	Any NAT2*4 of case	Non-NAT2*4 of control	Any NAT2*4 of control	NOS scores
Vilckova	2014	Slovak	Caucasian	HB	PCR-RFLP	395	281	172	109	230	165	8
Cox	2011	NM	Caucasian	PB	NM	9965	6,953	4,128	2,825	5,974	3,991	9
Kidd	2011	USA	African	HB	Taqman	493	190	78	112	201	292	7
Sharma	2010	Mixed	Mixed	PB	Taqman	2063	2,106	818	1,288	814	1,249	9
Iguchi	2009	USA	Caucasian	PB	PCR	170	180	111	69	92	78	8
Srivastava	2005	India	Caucasian	PB	PCR-RFLP	140	130	46	84	62	78	9
Costa	2005	Portugal	Caucasian	PB	PCR-RFLP	174	146	60	86	79	95	9
Rovito	2005	USA	Caucasian	PB	PCR	146	139	88	51	82	64	8
Gao	2003	China	Asian	PB	PCR-RFLP	112	58	13	45	20	92	9
**Hamasaki**	**2003**	**Japan**	**Asian**	**HB**	**PCR-RFLP**	**152**	**111**	**19**	**92**	**13**	**139**	**7**
Hein	2002	USA	Caucasian	HB	PCR-RFLP	115	47	31	16	60	55	8
**Wang**	**2002**	**Poland**	**Caucasian**	**HB**	**PCR-RFLP**	**17**	**34**	**21**	**13**	**5**	**12**	**8**
Wadelius	1999	Sweden	Caucasian	PB	PCR	519	331	202	129	320	199	9
Agundez	1998	Spain	Caucasian	PB	PCR	160	94	52	42	83	77	8

Abbreviations: SOC: Source of controls; PB: Population-based controls; HB: Hospital-based controls; NM: not mentioned; NOS:Newcastle-Ottawa-Scale.

Any NAT2*4:Rapid acetylation; Non-NAT2*4:Slow acetylation.

Notes: The studies of Hamasaki et al and Wang et al (shown in bold) were removed later because of its heterogeneity and publication bias.

### Quantitative synthesis results

**Figure 2:**
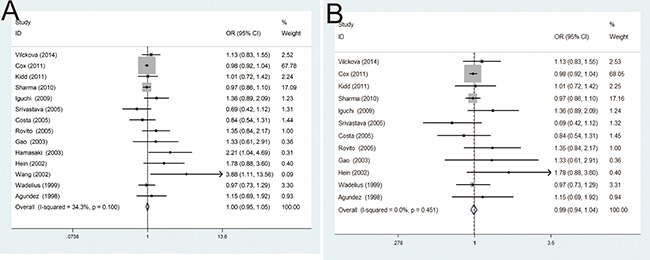
(**A**) Forest plots of the association between NAT2 polymorphism and PCa susceptibility in fixed model; (**B**) Forest plots of the association between NAT2 polymorphism and PCa susceptibility in fixed model after omitting two studies by Hamasaki et al. and Wang et al. with heterogeneity and publication bias.

Overall, the main results of this meta-analysis about the associations between NAT2 polymorphism and PCa risk were shown in Table 2. Initially, the results from this meta-analysis indicated that there was no significant susceptibility for the NAT2*4 allele with PCa risk (OR = 1.00, 95% CI = 0.95–1.05; *P* = 0.100). However, in order to find possible factors, heterogeneity analysis and publication bias were tested. After excluding two studies of Hamasaki et al. and Wang et al. in the existence of heterogeneity and publication bias, the results demonstrated that no significant relationship was also found between NAT2*4 allele and the risk of PCa, in fixed-effects model (OR = 0.99, 95% CI = 0.94–1.04; *P* = 0.451) (Figure 2).

**Table 2:** Meta-analysis results of association between NAT2 polymorphism and prostate cancer risk after the elimination of the two studies by Hamasaki et al and Wang et al

	N^a^	Sample Size	OR (95% CI)*	P^b^
Total	12	25,107	0.99 (0.94, 1.04)	0.451
Ethnicity				
Caucasion	9	20,085	0.99 (0.94, 1.05)	0.245
Genotyping				
PCR–RFLP	5	1,598	1.02 (0.83, 1.26)	0.169
Taqman	2	4,852	0.98 (0.87, 1.10)	0.839
PCR	4	1,739	1.13 (0.93, 1.37)	0.505
Source of control				
HB	9	1,521	1.13 (0.91, 1.41)	0.371
PB	3	23,586	0.98 (0.93, 1.03)	0.488

^a^Number of studies.

^b^*P* value of *Q* test for heterogeneity.

*Random-effects model was used when *P* value for heterogeneity test < 0.05; otherwise, fixed-effects model was used.

**Figure 3:**
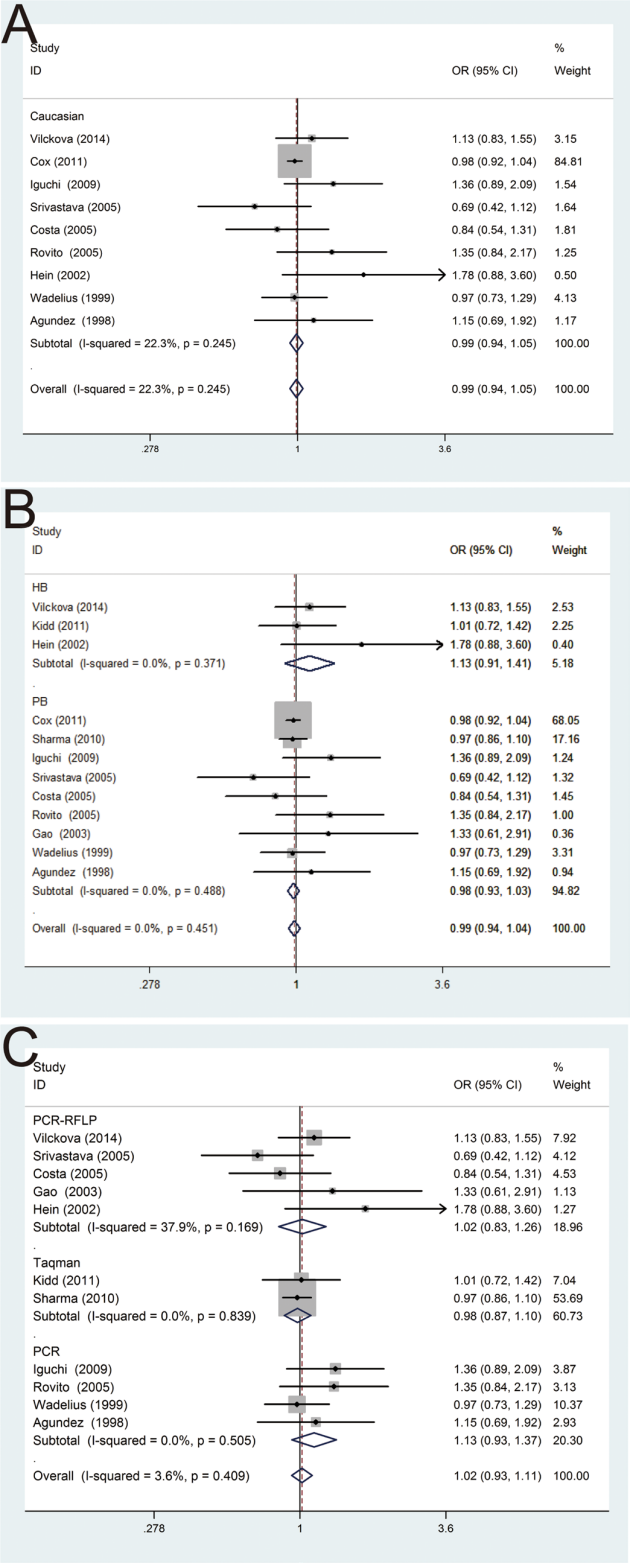
Forest plots of subgroup analysis of the association between NAT2 polymorphism and PCa susceptibility in fixed model (**A**) stratified by ethnicity; (**B**) stratified by source of controls; (**C**) stratified by genotyping methods.

In the subgroup analysis by ethnicity, the results were no statistical significance in Caucasian population (OR = 0.99, 95% CI = 0.94–1.05) (Figure 3A). Moreover, subgroup analysis by control source groups were also performed, and no statistically significant results were detected in population-based control group (OR = 0.98, 95% CI = 0.93–1.03) and hospital-based control group (OR = 1.13, 95% CI = 0.91–1.41) (Figure 3B). In addition, in the subgroup analysis by different genotyping methods, no significant results of such association were found in TaqMan (OR = 0.98, 95% CI = 0.87–1.10), PCR (OR = 1.13, 95% CI = 0.93–1.37), PCR-RFLP (OR = 1.02, 95% CI = 0.83–1.26), respectively (Figure 3C). In general, there was no association between NAT2 polymorphism and PCa risk in the comparisons of without NAT2*4 and with NAT2*4.

### Publication bias

**Figure 4:**
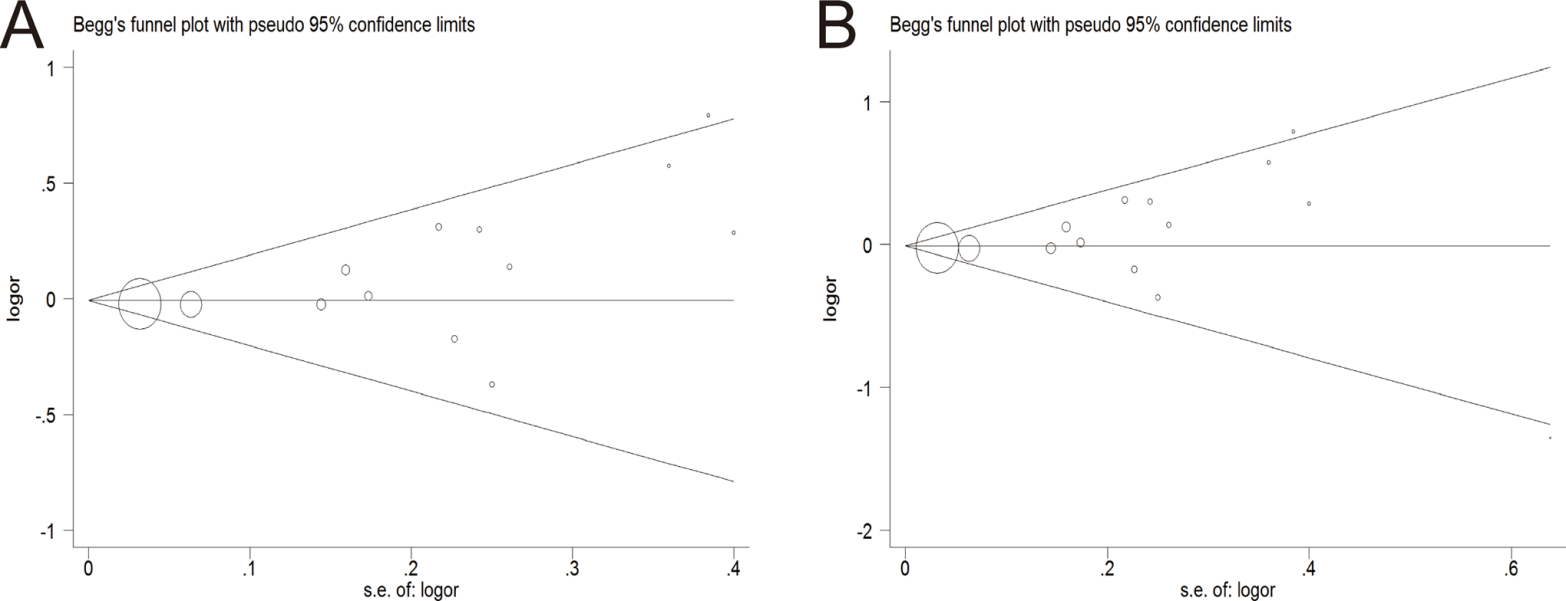
Begg's funnel plot of publication bias test (**A**) Before omitting a study of Hamasaki et al. (**B**) After the exclusion of the study.

The Egger's test and the Begg's funnel plot were used to assess the potential publication bias. Before excluding two studies by Hamasaki et al. and Wang et al., the plots exhibited the potential publication bias, because the plots were asymmetric in the Begg's funnel (Figure 4A). Nevertheless, after eliminating this study, the shapes of the funnel plots seemed symmetrically distributed, indicating little evidence of significant publication bias across studies (*P* = 0.131) (Figure 4B).

### Test of heterogeneity

**Figure 5:**
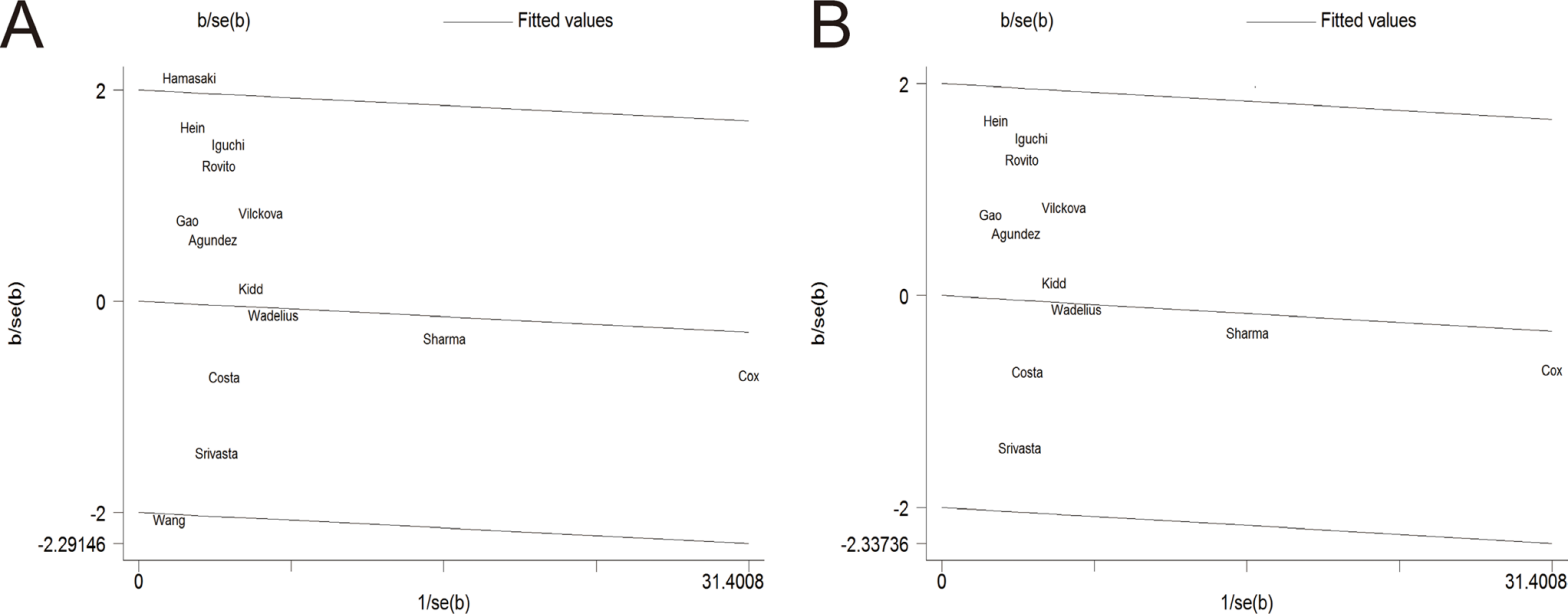
Galbraith plot of the association between NAT2 polymorphism and PCa susceptibility in fixed model (**A**) Before removing a study by Hamasaki et al. (**B**) After the exclusion of the study.

After excluding two studies by Hamasaki et al. and Wang et al., the overall heterogeneity was obviously decreased (*P* = 0.451), which indicated that two studies by Hamasaki et al. and Wang et al. might have generated the origin of heterogeneity (Figure 5). What's more, it was interesting that subgroup analyses were performed to reduce the heterogeneity.

### Trial sequential analysis results

**Figure 6:**
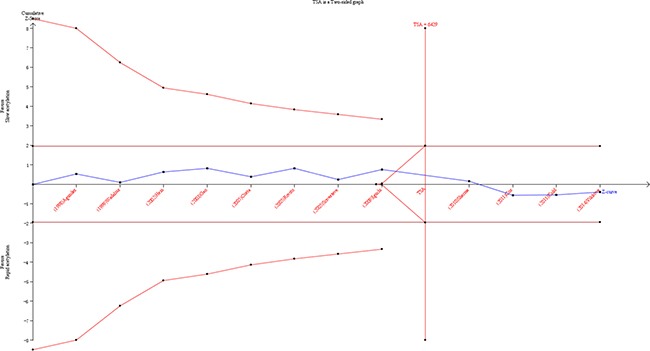
Trial sequential analysis of the association between NAT2 polymorphism and the risk of PCa The required information size was calculated based on a two side α = 5%, β = 15% (power 85%), and a relative risk reduction of 20%.

As shown in Figure 6, although the cumulative Z-cure didn't cross the trial sequential monitoring boundary, the total number of cases and controls were more than the required information size. To sum up, the sufficient evidence of our results were established and further relevant trials were unnecessary.

## DISCUSSION

The NAT2 gene is located on chromosome 8p21.3-23.1 and encodes a 290-amino-acid protein [35, 36]. As an essential phase II enzyme, NAT2 is key in the process of cancer development. Besides, this gene is essential in the metabolism of aromatic heterocyclic amines and hydrazines via N- and O-acetylation [37]. Alterations to the NAT2 acetylator status caused by variations in the NAT2 gene have been reported to reduce enzymatic activity, resulting in inefficient detoxification and thus leading to increased cancer susceptibility. It has been reported that the variant alleles in NAT2 lead to slow elimination of carcinogenic amines. Two major NAT2 phenotypes have been classified, such as rapid and slow. Several NAT2 genetic variants have been identified in human being, of which NAT2*4 is regarded as the most common allele associated to rapid acetylation. The rapid acetylator phenotype was in association with increased risk of colon, bladder, and PCa [38–40]. Whereas slow acetylator phenotype was reported to the decreased risk of colon cancer and increased the risk of bladder cancer [41, 42].

A small number of studies have investigated the involvement of NAT2 gene in the etiology of PCa, but they have ultimately led to conflicting results. Therefore, no firm conclusion has been provided regarding NAT2 gene role in PCa risk. On the one hand, the finding by Hamasaki et al. showed that NAT2 slow acetylator genotype increased the risk of PCa in Japanese patients, especially among smokers [14]. On the other hand, the result of another study by Costa S et al. suggested that NAT2*6E could be associated with the susceptibility of PCa [29]. What's more, another study by Srivastava et al. suggested no significant association between NAT2 genotype and PCa risk was found in the North Indian population, but this study reported an association between NAT2 rapid acetylator genotypes and tobacco users with PCa [28]. Hence, no unified conclusions have been provided regarding the role of NAT2 gene polymorphism in PCa risk. In our meta-analysis, we attempt to clarify whether or not the NAT2 polymorphism is correlated with the susceptibility of PCa. Besides, TSA was performed to effectively reduce the risk of type I error and assess whether the evidence of our results was reliable.

Meta-analysis is a powerful tool, which can make the conclusion more credible compared with a single study, especially in analyzing unexplained associations [43]. As the development of the current meta-analysis, we performed it to provide the more comprehensive understanding of the relationship between NAT2*4 allele and the risk of PCa by different subgroup analysis. As a consequence, we took advantage of meta-analysis to explain this possible association. In the present meta-analysis, 13 independent case-control studies were included with a total of 14,469 PCa patients and 10,689 controls. Our results revealed that no significant relationship was detected between NAT2*4 allele and the increased risk of PCa. This contradictory could be caused by several factors, including the differences in sample size, genotyping method, study design, and statistical method and so on.

Several subgroup meta-analyses were performed according to different ethnic groups, different source of controls and different methodologies. In racial subgroups, there was no association between NAT2*4 allele and the risk of PCa appeared in Caucasians. Nevertheless, the result might not be very conclusive, because of the relatively small number of African-derived and Asian-derived populations used in the meta-analysis. What's more, because of a mixture population from different geographic regions and other races, there was a significant between-study heterogeneity in Caucasians, which might lead to the negative results of our analysis.

In the subgroup analysis by source of controls, no statistical significance about such association was also observed in both population-based control group and hospital-based control group. Besides, there were healthy population and other disease patients with the exception of PCa in the controls included. It was likely that different individuals in the control group might have different risk of developing PCa, thus to affect the quality of the studies.

After stratified analysis by genotyping method, no statistically significant increased PCa risk was shown in TaqMan, PCR, PCR-RFLP and so on. Different genotyping methods had their own benefits in various aspects, which might result in different statistical results. Thus, applying the same appropriate genotyping method might make the meta-analysis results more significance and reliable. What’ more, it is necessary to have a unified admission criteria and a larger sample size of the relevant researches.

As an useful tool, TSA is similar to interim analyses in a single trial, where monitoring boundaries are used to decide whether additional trials are needed to evaluate for evidence when a *P* value is sufficiently small to show the anticipated effect or for futility [44, 45]. As is well-known to all, traditional meta-analysis may result in type I and type II errors. However, TSA is performed to reduce the risk of type I error by estimation of required information size with an adjusted threshold for statistical significance, and estimate whether further trials are necessary. If the cumulative Z-curve crosses the trial sequential monitoring boundary or the required information size, it shows firm evidence for such study. If not, it is necessary to do an additional clinical trial to reach a consistent conclusion [19, 20, 21]. As shown in our study, the cumulative Z-curve has reached the perpendicular line (required information size). Therefore, the evidence of the result is sufficient to reach a conclusion.

Notably, we included more studies with the large sample size to estimate a slight association by this meta-analysis, and this is the first TSA to comprehensively illustrate the impact of NAT2 polymorphism in response to PCa risk. However, several limitations should be taken into consideration. Firstly, the sample size in each stratified analyses was relatively small and might potentially limit the enough statistical power to explore the real relationship. Therefore, further studies with a larger sample size were still needed to be further validated. Secondly, the pathogenesis of PCa, as a multi-factorial disease, is closely related to environmental backgrounds as well as the interaction with various genetic factors instead of the influence of any single gene. Therefore, gene-to-environment interactions have been an important role to evaluate genetic polymorphisms. Further studies and more original data are needed to evaluate potential gene-to-gene and gene-to-environment interactions. In addition, we couldn't get useful data about the association between NAT2 polymorphism and the risk of PCa in the GWAS database. Although we could not obtain useful data in the GWAS database, a large number of articles related to NAT2*4 allele with PCa risk were found. What's more, a certain deviation may be resulted by a combined analysis of researched population in different ages, ethnicities and types of PCa. Thereby, this risk factor may cause a certain heterogeneity. Additionally, the incidence of PCa was different among different races. The majority studies included were investigated in Caucasian population in this meta-analysis. Therefore, the outcome of ethnic sub-group analysis might be affected.

## MATERIALS AND METHODS

### Literature search

We searched the relevant studies by electronic database PubMed, EMBASE and Web of Science, with the last search update on March 1st, 2017. The combination of the following terms were used: “N-acetyltransferase-2” or “NAT2”, “single nucleotide polymorphism” or “variants”, or “polymorphism”, and “prostate cancer” or “prostate neoplasm” or “prostate tumor”. In addition, we also screened by a manual search from the references of the original articles and retrieved articles considered eligible for the meta-analysis. Moreover, only the latest or more comprehensive study was selected in this meta-analysis, if studies had partly overlapping or familiar subjects.

### Inclusion and exclusion criteria

Eligible studies were selected according to the following criteria: (a) An independent case-control design; (b) The relationship between NAT2 gene polymorphism and susceptibility to PCa was evaluated; (c) The abundant data to evaluate the pooled odds ratios (ORs) with 95% confidence intervals (CIs) was provided.

Exclusion criteria from this meta-analysis were as follows: (a) Non-case-control studies; (b) Review articles; (c) No original available data of genotype frequency to evaluate this association; (d) Previous duplicated publications.

### Data extraction

Two investigators (Wang F and Qin ZQ) independently extracted the data from the identified studies. In addition, any disagreement was resolved by a discussion with a third reviewer until consensus was based on the main point of view. The following information were recorded in a standardized form: first author's last name, year of publication, country, ethnicity, source of controls (population-based or hospital-based), genotying method, number of cases and controls, and genotype frequency of NAT2 gene polymorphism between cases and controls, respectively.

### Quality assessment

The quality of the studies was assessed using the validated Newcastle-Ottawa Scale (NOS) for non-randomized studies, including case-control and cohort studies. A study can be awarded a maximum of one star for each point within the selection and exposure categories, and a maximum of two stars can be given for comparability. We considered studies with scores of more than 7 as high-quality studies and only high-quality studies were included in our meta-analysis.

### Statistical analysis

The pooled ORs with 95% CIs were calculated to assess the strength of association between NAT2*4 allele and PCa risk. Two models were used in the meta-analysis, including the fixed-effects model (a Mantel–Haenszel method) and the random-effects model (a DerSimonian–Laird method). If *P* values < 0.05, the random-effects model would be conducted; Otherwise, the fixed effects model would be used to perform meta-analysis. What's more, subgroup analysis was further carried out to explore the potential sources of heterogeneity according to ethnicity, source of controls and genotyping methods. Besides, the influence of publication bias between the studies was estimated by Begg's funnel plots and Egger's linear regression test, and *P* < 0.05 was considered to be statistically significant. All above statistical data were conducted by Stata software (version 12.0, Stata Corporation, College Station, TX).

### Trial sequential analysis

A cumulative meta-analyses with addition of new publishing trials might result in type I and type II errors, because it could increase the risk of random error with repeated significance testing and sparse data [15–17]. Therefore, TSA was introduced to reduce the risk of type I error, which could estimate the required information size with an adjusted threshold for statistical significance [18, 19]. In the current meta-analysis, TSA was performed by anticipating a 20% relative risk reduction for efficacy outcome, an overall 5% risk a type I error and a statistical test power of 80%, to estimate the required diversity-adjusted information size [20]. When the cumulative Z-curve crosses the trial sequential monitoring boundary, a sufficient level of evidence has been reached and further studies are unneeded. If the Z curve dose not cross any of the boundaries and the required information size has not been reached, it is needed to try an additional clinical trial to reach a sufficient conclusion [21]. In this study, we applied the trial sequential analysis software (TSA, version 0.9; Copenhagen Trial Unit, Copenhagen, Denmark, 2011).

## CONCLUSIONS

The results of this meta-analysis demonstrated that no evidence supporting the relationship between NAT2 polymorphism and PCa risk was detected. More importantly, further studies in other ethnic groups, such as Asians, Africans, are needed to give more comprehensive understanding of such association.
